# Novel NUDCD1 gene variant predisposes to severe COVID-19 disease in Asians through modulation of antiviral DHX15- and MAVS-mediated signalling

**DOI:** 10.3389/fimmu.2025.1581293

**Published:** 2025-06-04

**Authors:** Aseervatham Anusha Amali, Douglas Jie Wen Tay, Yiqi Seow, Marie Loh, Sharada Ravikumar, Jocelyn Jin Yu, Shaun Seh Ern Loong, Siew Wai Fong, Chang Jie Mick Lee, Jonathan Jordon Cailu Lim, Louis Hanqiang Gan, Winston Lian Chye Koh, Ying Ding, Qi Hui Sam, Zhaohong Tan, Rachel Ying Min Tan, Chong Boon Lua, Justin Jang Hann Chu, Amit Singhal, Shyam Prabhakar, Wee Joo Chng, Laurent Renia, David Chien Boon Lye, Lisa F. P. Ng, Kai Sen Tan, Roger Foo, Chang Chuan Melvin Lee, Barnaby Young, Louis Yi Ann Chai

**Affiliations:** ^1^ Division of Infectious Diseases, Department of Medicine, National University Health System, Singapore, Singapore; ^2^ Infectious Diseases Translational Research Programme and Department of Microbiology and Immunology, Yong Loo Lin School of Medicine, National University of Singapore, Singapore, Singapore; ^3^ Biosafety Level 3 Core Facility, Yong Loo Lin School of Medicine, National University of Singapore, Singapore, Singapore; ^4^ Genome Institute of Singapore, Agency for Science, Technology and Research (ASTAR), Singapore, Singapore; ^5^ Lee Kong Chian School of Medicine, Nanyang Technological University, Singapore, Singapore; ^6^ Department of Epidemiology and Biostatistics, School of Public Health, Imperial College London, London, United Kingdom; ^7^ Infectious Diseases Research Laboratory, National Centre for Infectious Diseases, Singapore, Singapore; ^8^ Cardiovascular Research Institute, Yong Loo Lin School of Medicine, National University of Singapore, Singapore, Singapore; ^9^ ASTAR Infectious Diseases Labs (ASTAR ID Labs), Agency for Science, Technology and Research (ASTAR), Singapore, Singapore; ^10^ Bioinformatic Institute, ASTAR (Agency for Science, Technology and Research), Singapore, Singapore; ^11^ Department of Anaesthesia, National University Health System, Singapore, Singapore; ^12^ Collaborative and Translation Unit for Hand, Foot and Mouth Disease (HFMD), Institute for Molecular and Cell Biology, Agency for Science, Technology and Research (ASTAR), Singapore, Singapore; ^13^ Department of Hematology-Oncology, National University Cancer Institute of Singapore (NCIS), National University Health System, Singapore and Cancer Science Institute of Singapore, National University of Singapore, Singapore, Singapore; ^14^ Department of Medicine, Yong Loo Lin School of Medicine, National University of Singapore, Singapore, Singapore; ^15^ Department of Biochemistry, Yong Loo Lin School of Medicine, National University of Singapore, Singapore, Singapore; ^16^ Rural Clinical School, Toowoomba Regional Clinical Unit, The University of Queensland, Brisbane, QLD, Australia

**Keywords:** SARS-CoV-2, innate immunity, DEAH-Box helicase, type I interferon, Southeast Asia

## Abstract

**Background:**

Genome-wide associative studies can potentially uncover novel pathways which modulate anti-viral immune responses against SARS-CoV-2 or identify drivers of severe disease. To date, these studies have yielded loci mostly in non-functional domains of unknown biological significance and invariably require large sample sizes, potentially missing lower frequency variants, especially in under-represented or minority populations.

**Methods:**

To identify unique genetic traits predisposing to severe COVID-19 in Asians, we employed an alternative strategy using whole exome sequencing of representative cohort of severe versus mild COVID-19 patients. Candidate gene variants were identified by performing logistic regression against top genetic principal components, prioritised for missense variants with likely causal impact. Then, functional sequelae of variants were replicated *in-vitro* and re-validated in patients ex vivo to demonstrate causality between genotype and clinical phenotype.

**Results:**

Of 136 COVID-19 patients in Singapore (of whom 25% had severe disease), a single nucleotide polymorphism rs2980619 (p.L252F substitution) belonging to NudC-Domain-Containing-1 (NUDCD1) was highly-placed. Homozygous bearers of variant p.L252F had higher (3.97x) odds of severe disease. Age >50 years and male sex were significant covariates which increased the odds of severe disease by 3.38x and 3.16x, respectively. We showed *in-vitro* that variant p.L252F reduced NUDCD1 activity, leading to reduced antiviral signalling through RNA helicase DHX15 and antiviral signalling adaptor MAVS, reduced activation of NFκB components RelB and p65, and resultant 1-log higher SARS-CoV-2 viral load compared to wild type (L252) cells. Patients bearing p.L252F had lower NUDCD1, MAVS, and RelB expressions, affirming the above findings.

**Conclusion:**

A gene variant of NUDCD1 influences COVID-19 severity in Asians through interacting with DHX15 and MAVS, affecting effective response against SARS-CoV-2.

## Introduction

COVID-19 disease manifestations are protean, from asymptomatic infection to severe disease with organ failure. Amongst the clinical factors identified, age confers the clearest risk for severe disease whereby patients >65 years old are at 5x higher risk of hospitalisation and 65x higher risk of death, alongside ethnicity, chronic kidney and lung diseases, and malignancy (1). However, through the 3 years of the pandemic, we had seen younger patients who were previously well, with COVID-19 who required supplemental oxygen or mechanical ventilation in the ICU; conversely, elderly SARS-CoV-2-infected patients with ailments had passed through relatively unscathed. These observations point to additional contributions by intrinsic host traits which dictate susceptibility to the SARS-CoV-2 virus.

To date, genome wide association studies (GWAS) involving tens of thousands of subjects have identified more than 20 loci associated with increased susceptibility or severity to SARS-CoV-2 infection (as described in **Table 1**). The majority of these loci lie in purported non-functional domains which, despite some showing robust association, have unascertained biological significance. GWAS utilises pre-established genotypic database (derived primarily from Caucasian cohorts) to measure association between disease phenotype and gene variant. However, the strength of the computed association can be compromised as allele frequencies decrease. Furthermore, GWAS can miss yet-to-be-identified novel variants (e.g. in Asians) not incorporated into existing genotyping arrays (18), as exemplified by the recent identification of DOCK2 (dedicator of cytokinesis 2) in the Japanese COVID-19 cohort (11). Whole exome or genome sequencing can enhance the detection of rare variants. Studying the effect of low frequency or rare variants invariably requires large sample sizes, often running into tens of thousands lest the study risks being under-powered. The requirement of such large numbers for GWAS necessitates incorporation of multi-national cohorts as extraordinarily exemplified by the COVID-19 Host Genetics Initiative, which recently identified the TLR7 gene amongst others as complicit in severe COVID-19 (19). In such a trans-national setting, however, minority ethnic-specific predisposing traits may be missed, as has been exemplified by the COVID-19 Host Genetics Initiative whereby East Asians constitute only 10% of the overall study population (20).

**Table 1:** Published genetic studies examining susceptibility to severe COVID-19 disease as a whole or part of the study.

Study (Year)	Participants	Controls	Population / Genetic ancestry	Sequencing methodology	Genetic cluster(s)	Lead SNP(s) or variant	Genes implicated	Mechanistic study performed
Severe COVID 19 GWAS group (2020) (2)	Severe COVID-19 defined as respiratory failure requiring supplemental oxygen or mechanical ventilation *n* = 1610	Healthy volunteers, blood donors, and outpatients* *n* = 2205	European – Italy, Spain	GWAS	3p21.31 9q34.2	rs11385942 rs657152	*LZTFL1, CXCR6, SLC6A20*, *CCR9, FYCO1, XCR1* *ABO*	No
Pairo-Castineira E, Clohisey S, Klaric L, et al. (2020) (3)	Critically ill COVID-19 patients *n* = 2,244	Population controls from population genetic studies in the United Kingdom (UK) (UK Biobank) *n* = 5 ancestry-matched controls per participant	Mixed – Europeans, South Asians, Africans, East Asians (East Asian 149 [6.6%], South Asian 237 [10.5%])†	GWAS	12q24.13 19p13.2 19p13.3 21q22.1	rs10735079 rs74956615 rs2109069 rs2236757	*OAS1, OAS2, OAS3* *TYK2* *DPP9* *IFNAR2*	No
Kousathanas A, Pairo-Castineira E, Rawlik K, et al. (2020) (4)	Critically ill COVID-19 patients *n* = 7,491	Population controls (100,000 Genomes Project) and mild COVID-19 *n* = 48,400 (100,000 Genomes Project cohort, *n* = 46,770; mild COVID-19, *n* = 1,630)	Mixed – East Asian 274/366 (3.7%/0.8%); South Asian 788/3,793 (10.5%/7.8%)	WGS	1q.21.1 1q.22 1q.22 2p16.1 3p21.31 3p21.31 3q24 5q31.1 6p21.1 6p21.32 9p21.3 11p13 12q24.33 13q34 15q26.1 16q24.3 17q21.31 17q21.33 19p13.2 19p13.2 19p13.3 19q13.33 21q22.11 21q22.11 21q22.11	rs114301457 rs7528026 rs41264915 rs1123573‡ rs2271616 rs73064425 rs343320 rs56162149 rs2496644 rs9271609 rs28368148 rs61882275 rs56106917 rs9577175 rs4424872 rs117169628 rs2532300 rs3848456 rs73510898 rs34536443 rs12610495 rs368565 rs17860115 rs8178521 rs35370143	*EFNA4* *EFNA1, TRIM46* *THBS3* *BCL11A‡* *SLC6A20* *LZTFL1* *PLSCR1* *CSF2, ACSL6* *LINC01276* *HLA-DRB9, HLA-DRB1* *IFNA10* *ELF5* *FBRSL1* *ATP11A* *RGMA* *SLC22A31* *CRHR1, KANSL1* *-* *TYK2, ZGLP1* *TYK2* *DPP9* *FUT2* *IFNAR2* *IL10RB* *LINC00649*	No
Shelton JF, Shastri AJ, Ye C, et al. (2021) (5)	Severe COVID-19 participants receiving either respiratory support or who had pneumonia *n* = 1,447	Participants who did not report a COVID-19 diagnosis *n* = 796,151	Mixed – no Asian participants	GWAS	3p21.31	rs13078854	*LZTFL1 SLC6A20, CCR9, FYCO1, CXCR6, XCR1*	No
	COVID-19 participants receiving support in the form of supplementary oxygen or ventilation *n* = 636	Participants who did not report a COVID-19 diagnosis *n* = 797,180						
	COVID-19 participants who had pneumonia *n* = 1,286	Participants who did not report a COVID-19 diagnosis *n* = 797,084						
	COVID-19 participants who were hospitalised for symptoms *n* = 802	Participants who did not report a COVID-19 diagnosis *n* = 797,153						
Wu P, Ding L, Li X, et al. (2021) (6)	Severely ill COVID-19 patients *n* = 598	Population controls and mild COVID-19 patients *n* = 2,260 (control = 1.401; mild COVID-19 = 859)	East Asian – Chinese	GWAS§	3p21.31 6p.21.1 9q43.2 19q13.11¶	rs35044562 rs1853837 rs8176719 rs74490654¶	*LZTFL1* *FOXP4-AS1* *ABO* *MEF2B*¶	No
	Severely ill COVID-19 patients *n* = 474	Population controls and mild COVID-19 patients *n* = 1,615 (control = 948; mild COVID-19 = 667)		WGS§				
Li Y, Ke Y, Xia X, et al. (2021) (7)	Severe or critically ill COVID-19 patients *n* = 885	Mild or moderate COVID-19 patients *n* = 546	East Asian – Chinese	GWAS	11q14.2 11q23.3	rs10831496 rs1712779	*CTSC* *NNMT, REXO2, CADM1*, *C11orf71*	No
Gong B, Huang L, He Y, et al. (2022) (8)	Critically ill COVID-19 patients *n* = 632	Healthy controls *n* = 3,021	East Asian – Chinese	GWAS	2p12 2p14 2q24.3 3p25.2 7p15.3 7q21.11	rs72809129 rs10519086 rs7422259 rs7598285 rs2069837 rs17158686	*LRRTM4* *LINC01799* *SCN7A* *LINC01247* *IL6* *SEMA3A*	Yes – measurements of serum IL-6 levels and relative luciferase activity
Zecevic M, Kotur N, Ristivojevic B, et al. (2022) (9)	Moderate and severely ill COVID-19 patients *n* = 80 (moderate = 46, severe = 34)	Mild COVID-19 patients *n* = 48	Serbian	GWAS	3p21.31 3p21.31 5q11.2 5p15.33 9p23 13q21.33	rs73060324 rs35280891 rs78317595 rs13176661 rs1331359 rs61964606	*SACM1L* *LZTFL1* *ESM1* *IRX4, NDUFS6, MRPL36* *TYRP1, LURAP1L* *KLHL1, ATXN8, ATXN80S*	No
Pandit R, Singh I, Ansari A, et al. (2022) (10)	Deceased COVID-19 patients *n* = 60	Recovered COVID-19 patients *n* = 228	South Asian - Indian	GWAS	1q25.1 4p15.2 4p15.2 5q14.3 10p12.1 11p14.2 13q12.11 14q24.3 14q32.31 14q32.31 16p12.3 16p12.3 16p12.3	rs17300100 rs73246461 rs12651262 rs4424029 rs12773860 rs10835056 rs398077102 rs34279101 rs12323812 rs11160678 rs1453512 rs1597988 rs4371135	*TNFSF4, TNFSF18, RP1–15D23.2, GOT2P2* *PPARGC1A, DHX15* *DHX15, RN7SL16P, PPARGC1A* *CTD, 2036A18.2, PTP4A1P4* *ANO3, SLC5A12* *WAC-AS1, BAMBI, WAC-AS1, RNU4ATAC6P, TPRKBP1, RNU6,1067P, snRNA* *LINC00540, AL354828.1* *ANGEL1, VASH1-AS1, AC007376.2, RN7SKP17, misc_RNA, AF111169.1, LRRC74A, RPL22P2, AF111169.4* *MOK, CINP, TECPR2, ZNF839* *MOK, TECPR2, ZNF839, CINP* *AC098965.1* *AC098965.1* *AC098965.1*	No
	Deceased COVID-19 patients *n* = 60	Asymptomatic COVID-19 patients *n* = 111						
Namkoong H, Edahiro R, Takano T, et al. (2022) (11)	Severely ill COVID-19 patients requiring oxygen support, artificial respiration, and / or intensive care *n* = 990 (440 aged < 65 years)	Population controls recruited prior to the COVID-19 pandemic *n* = 3,289 (2,377 aged < 65 years)	East Asian – Japanese	GWAS	3p21.31 5q35.1** 6p21.1 9q34.2 17q21.33 19p13.3 21q22.11	rs35081325 rs60200309** rs1886814 rs529565 rs77534576 rs2109069 rs13050728	*LZTFL1* DOCK2** *FOXP4* *ABO* *TAC4* *DPP9* *IFNAR2*	Yes – histopathology, viral loads, and cytokine expression assays in a Syrian hamster model
	Severely ill COVID-19 patients requiring oxygen support, artificial respiration, and / or intensive care *n* = 1,243††	Population controls, details unspecified *n* = 3,769 (1,242 aged < 65 years)						
Pereira AC, Bes TM, Velho M, et al. (2022) (12)	Hospitalised COVID-19 patients *n* = 3,533	Non-hospitalised COVID-19 participants	European –Brazilian	GWAS	1q32.1	rs11240388	*RBBP5, DSTYK, TMCC2*	No
Cruz R, Diz-de Almeida S, López de Heredia M, et al. (2022) (13)	Critically ill COVID-19 patients *n* = 1,128	Non-severe COVID-19 patients and population controls with unknown COVID-19 status *n* =16,754	European –Spanish	GWAS	3p21.31 3p21.31 3p21.31 3p21.31 3p21.31 9p13.3 17q21.31 19q13.12 21q22.11 9q21.32‡‡	rs115679256 rs17763742 rs35477280 rs4443214 rs115102354 rs10813976 rs1230082 rs77127536 rs17860169 rs140152223‡	*LIMD1* *LZTFL1* *FYCO1* *XCR1* *CCR3* *AQP3* *ARHGAP27* *UPK1A/ZTBT32* *IFNAR2* *TLE1*‡‡	No
	Severely ill COVID-19 patients *n* = 2,379	Non-severe COVID-19 patients and population controls with unknown COVID-19 status *n* = 14,375						
	Hospitalised COVID-19 patients *n* = 5,966	Non-hospitalised COVID-19 patients and population controls with unknown COVID-19 status *n* = 11,916						
	COVID-19 patients *n* = 11,939	Population controls with unknown COVID-19 status *n* = 5,943						
COVID-19 Host Genetics Initiative (meta-analysis of 46 studies, including the update following data release 6) (2021,2022) (14, 15)	Critically ill COVID-19 patients requiring respiratory support or who died as a result of COVID-19 *n* = 9,376	Population controls with unknown COVID-19 status *n* = 1,776,645	Mixed – exact Asian proportion not reported (19 countries)	GWAS§§	1p21.3 3p21.31 3p21.31 6p21.33 6p21.1 9q34.2 10q22.3 11p15.5 11p13 12q24.13 12q24.33 16q24.3 17q21.31 17q21.33 19p13.3 19p13.3 19q13.2 21q22.1	rs67579710 rs73062389 rs35508621 rs111837807 rs1886814 rs912805253 rs721917 rs35705950 rs766826 rs10774679 rs12809318 rs117169628 rs61667602 rs77534576 rs2109069 rs74956615 rs1405655 rs13050728	*MUC1, THBS3, TRIM46* *SLC6A20, SACM1L* *LZTFL1, CXCR6* *HLA-B, HLA-C, CCHR1* *FOXP4* *ABO* *SFTPD* *MUC5B* *ELF5* *OAS1, OAS2, OAS3* *FBRSL1* *SLC22A31, ACSF3* *ARHGAP27, PLEKHM1* *KAT7, TAC4* *DPP9* *TYK2* *NAPSA, NR1H2* *IFNAR2, IL10RB*	No
	Moderate or severe COVID-19 patients hospitalised due to associated with COVID-19 infection *n* = 25,027	Population controls with unknown COVID-19 status *n* = 2,836,272						
Horowitz JE, Kosmicki JA, Damask A, et al. (2022) (16)	Severe COVID-19 patients *n* = 2,184	Non-hospitalised COVID-19 patients *n* = 45,185	Mixed – Africans, East Asians, Europeans, Latin Americans, South Asians (720 [1.5%])	GWAS	**3p21.31** 3p21.31 **6p21.33** 9q34.2 **19p13.3** **21q22.11**	**rs73064425** rs2531743 **rs143334143** rs879055593 **rs2109069** **rs2236757**	LZTFL1 SLC6A20 MHC ABO DPP9 IFNAR2	No
	Hospitalised COVID-19 patients *n* = 6,911	Non-hospitalised COVID-19 patients *n* = 45,185						
	Severe COVID-19 patients *n* = 2,184	Negative controls and unknown COVID-19 status *n =* 689,620						
	Hospitalised COVID-19 patients *n* = 6,911	Negative controls and unknown COVID-19 status *n =* 689,620						
	COVID-19 positive patients not hospitalised *n* = 45,641	Negative controls and unknown COVID-19 status *n =* 704,016						
	COVID-19 positive patients *n* = 52,630	Negative controls *n* = 109,605						
	COVID-19 positive patients *n* = 52,630	Healthy controls and unknown COVID-19 status *n =* 689,620						
Degenhardt F, Ellinghaus D, Juzenas S, et al. (2022) (17)	COVID-19 patients hospitalised with respiratory support (supplementary oxygen or mechanical ventilation) *n* = 3,255	Unknown COVID-19 status *n* = 12,488	European – Italy, Spain, Norway and Germany / Austria	GWAS	3p21.31 4p15.1 9p22.3 9q34.2 9q34.2 13q21.1 17q21.31 18p11.23 19p13.2 19p13.3 21q22.11	rs35731912 rs12512667 rs7023573 rs687289 rs550057 rs111671068 rs8065800 rs17565758 rs11085725 rs12610495 rs2834161	LZTFL1 PCDG7 FREM1 ABO ABO OLFM4 MAPT PTPRM TYK2 DPP9 IFNAR2	No
	Critically ill COVID-19 patients with mechanical ventilation *n* = 1,911	Unknown COVID-19 status *n* = 12,226						

COVID-19, Coronavirus Disease 2019; GWAS, Genome Wide Association Study; WGS, Whole Genome Sequencing.

* 40 healthy controls had evidence of SARS-CoV-2 antibodies with no or mild COVID-19 symptoms.

† Results in the East and South Asian ancestry groups unreliable due to high levels of residual inflation.

‡ No credible sets inferred using Bayesian fine mapping (posterior inclusion probability < 0.95).

§ No overlap between GWAS and WGS samples.

¶ Chinese-specific rare variant.

** Only in severely ill COVID-19 patients aged < 65 years-old.

†† Replication study conducted following additional recruitment.

‡‡ Associated with hospitalisation risk in females only with sex-disaggregated analysis.

§§ GWAS meta-analysis presenting 3 different phenotypes.

Acknowledging the challenges faced studying genetic predisposition in minority cohorts and in a resource- or cost-limited setting, one practical strategy would be to sequence a representative study cohort and validate the findings through mechanistic immunological and virological studies. To build upon findings from molecular epidemiological associative studies, it is imperative to replicate the functional sequelae of genotypic variation in experimental studies. This helps strengthen and demonstrate causality between genotype and clinical phenotype (21). This will be critical to our understanding to discover new pathways underlying the pathological processes against SARS-CoV-2 or drivers of severe disease state. We set about using this strategy to identify novel immune traits predisposing to severe COVID-19 in patients in Singapore.

## Methods

### Patient recruitment

Patients were recruited from the National Centre for Infectious Diseases, Singapore. We obtained 136 COVID-19 patient DNA specimens, of whom 25% had severe disease as defined by requiring oxygen or ventilation in ICU. The study protocols 2012/00917 and 2020/00435 were approved by the Domain Specific Research Board of National Healthcare Group, Singapore.

### Whole exome sequencing (WES)

DNA concentration and purity were quantified after extraction. 1µg of genomic DNA was fragmented to give a distribution of 200–500 base pairs. Library preparation was done using Kapa DNA HTP Library Prep Kit. Hybridisation of adapter ligated DNA was performed at 47°C, for 72h, to biotin-labelled probe included in the Nimblegen SeqCap EZ Human Exome Kit. Libraries were sequenced using the Illumina Hiseq4000 sequencing system and paired-end 151bp reads were generated for analysis.

### Bioinformatics analysis for WES

For genomic base calling, we followed best practice guidelines implemented in the GATK workflow (22). Raw sequencing reads from FASTQ files were aligned to the GRCh37 reference genome using the Burrows-Wheeler Aligner (BWA) program (23). Following alignment, Picard tools was used to label read groups and mark PCR duplicates to generate BAM files (24). The resulting BAM files were sorted and indexed. Local realignment around indels was performed to correct misalignments, and base quality score recalibration was applied to adjust the quality scores, aligning them more closely with the true probability of base mismatches in relation to the reference genome. Variant joint-calling was performed with HaplotypeCaller from GATK (version 4.5.0.0, GCCcore-12.3.0, Java-v17) using dbSNP reference b37, dbSNP version 137, with -ERC flag. Genotypes of individual chromosomes were called in parallel separately using GenomicsDBImport and GenotypeGVCFs functions to jointly aggregate multiple samples from previously single-sample haplotype calling. Following which, genotypes of all samples across all chromosomes were merged using the MergeVcfs function (22). Sites/variants were filtered by depth, base quality score, strand-bias and Hardy-Weinberg Equilibrium. High-quality variants were annotated using the Variant Effect Predictor program (25) to understand population-scale, ethnic-specific allele frequencies, variant-to-gene mappings, variant effect types, protein consequences and *in silico* functional consequence predictions. Identification of candidate gene was adopted from Ambry’s clinical variant classification scheme (26). We performed a logistic regression with oxygen requirement as end point in PLINK v1.9, adjusting for age and gender (27). Our sample size was powered to identify a minimum odds ratio (OR) of 1.8 at 80% power and 5% significance level. In view of the modest sample size and the intention to follow up with comprehensive laboratory confirmation, we did not apply multiple-testing correction for the significance level, but rather relied on filters by effect sizes (OR>1.8), minimum allele frequency (MAF) filters (MAF>0.4 across multiple ancestry populations) to ensure applicability for general population, and a preference for variants that associates with gene expression across multiple genes (number of expression quantitative trait loci [eQTL] > 5), which is more likely to represent a potential critical key role.

### Cloning and transfection

Candidate gene variants were amplified from human ORFeome v5.1 and cloned into pEGFP-C1 backbone using Gibson cloning with EGFP at the C-terminus. Mutant alleles were introduced by Gibson Assembly via overlapping primers targeting the mutant site (28). Mutant proteins were transfected into HEK293 cells to assay for expression and localisation, followed by SARS-CoV-2 infection in A549-ACE2 cells.

### Cell culture transfection and infection

A549-ACE2 cells were cultured in Dulbecco’s modified Eagle’s medium (DMEM; Gibco), supplemented with 10% foetal bovine serum (FBS) in 5% CO_2_ at 37°C. Cells were seeded into 24-well plates (ThermoFisher) at 10^5^ cells/well. Transfection of empty vector (EV), wild type NUDCD1 and NUDCD1 p.L252F variant were performed using Lipofectamine™ 3000 (Thermo Fisher Scientific) according to manufacturer’s protocol. After 48 hours of transfection, the cells were infected with wild type SARS-CoV-2 at MOI of 1 at 37°C with 5% CO_2_ in a BSL-3 facility. After 1h, the wells were topped up with 500µl of DMEM supplemented with 2% FBS and incubated for 48h. The supernatants were collected for plaque assay and ELISA.

### Plaque assay

Supernatants from infected cells were serially diluted 10-fold, added onto A549-ACE2 cells, and incubated for 1h at 37°C with 5% CO_2_. The inoculum was removed, overlaid with plaque medium containing 1.2% Avicel CL-611 (DuPont) with 2% FBS. After 3 days, the cells were fixed with 4% formaldehyde and stained with 0.5% crystal violet. Virus titre was calculated using the following formula:


$$\begin{array}{c} Viral\;titre\;\left(PFU/ml\right)\\ =\frac{Number\;of\;plaques}{Volume\;of\;inoculum\left(ml\right)}\times Dilution\;factor \end{array}$$

### Cytokine detection

ELISA for IL-6, TNFα and IFNα were performed according to manufacturer’s instructions (Invitrogen). Absorbance was measured with the Infinite M200PRO (Tecan) plate reader at 450 nm. The concentration of analytes was calculated from the respective standard curves and multiplied by the dilution factor.

### Western blotting

Infected cells were washed with ice-cold PBS and lysates collected at the indicated times post-infection with lysis buffer (1% NP-40, 2mM EDTA, 10% glycerol, 150mM NaCl, 50mM Tris HCl) supplemented with protease inhibitors (SigmaFAST) and phosphatase inhibitors (Roche PhosStop easy pack). Cell lysates were heat-inactivated at 65°C for 10min and protein concentration was determined by Bradford assay (Bio-Rad). Each 20μg of lysates was heated at 95°C for 5min, separated by SDS-PAGE (10%) and transferred to polyvinylidene difluoride membranes. Blots were blocked with 5% non-fat milk probed with following antibodies: NUDCD1 (1:500; Novus biologicals), DHX15 (1:500; sc271686; Santa cruz Biotechnology), anti-MAVS (1:500; A5764; ABclonal), anti-p65 (1:500; sc372; ScB), anti-RelB (1:1000; 10544S; Cell Signalling Technology), anti-IRF-3 (1:1000; D83B9; CST) and anti β-actin (1:1000; sc47778; ScB). Primary antibodies were incubated overnight at 4°C. Primary antibodies binding was detected using horseradish peroxidase-conjugated secondary antibodies (anti-rabbit, anti-mouse IgG, CST) by incubating at room temperature for 1h. Blots were visualised using Clarity Western ECL blotting substrate (Bio-Rad). The blots were stripped using Thermo Scientific Restore western blot stripping buffer (cat#21059) for reuse.

### Co-immunoprecipitation

Total proteins extracted after infection were used for co-immunoprecipitation and western blotting. DHX15 antibody was tagged with Dynabeads Protein G for magnetic selection and co-immunoprecipitated proteins were detected by western blotting using NUDCD1 and MAVS antibodies.

### Total RNA isolation and quantitative real-time PCR

RNA extraction was performed using TRIzol reagent (Invitrogen) according to manufacturer’s instructions. For cDNA synthesis, reverse transcription was carried out using iScript™ cDNA Synthesis Kit (Bio-Rad) and qPCR was performed using GoTaq qPCR Mastermix (Promega Corporation) in CFX connect thermocycler (Bio-Rad). Primer sequences for human NUDCD1 were: 5’-CTACTCTGGCAACCACACTCCA-3’ (forward) and 5’-CTCACAAAGGGCTGCATACGAG-3’ (reverse). Beta-2-microglobulin (B2M) was used as internal control: 5’-ATGAGTATGCCTGCCGTGTG-3’ (forward) and 5’-CCAAATGCGGCATCTTCAAAC-3’ (reverse). qPCR signals were quantified comparing cycle threshold (Ct) values of target gene with Ct values of reference gene B2M. Based on comparative Ct method, mean relative mRNA expression was calculated. The values were expressed as ratio of fold increase to control cell mRNA levels.

### RNA interference

The siRNA for NUDCD1 (hs.Ri.NUDCD1.13.1) was purchased from Integrated DNA Technologies. To inhibit NUDCD1 expression, cells were seeded (10^5^ cells/well) in 24-well plates in antibiotic-free medium. After 24h, the siRNA was transfected into cells using Lipofectamine™ 3000 Reagent (Thermo Fisher) according to manufacturer’s protocol. A scrambled (sc) siRNA (5’-AUGUUUGGAAGUGGAACCC-3’ and 5´-UAGGGUGUACCCGUAAUAG-3´) was used as negative control.

### Modeling of NUDCD1 docking

The most probable site of docking was determined using diffdock 4 https://github.com/gcorso/DiffDock). From the range of possible results, the result that had most highly probable structure across 10 runs was selected as input for PyMOL (https://pymol.org/2/) (29). PyMOL visualisation settings were set to showcase the possible hydrophobic and electrostatic interactions at the most possible docking site.

### Statistical analysis

Statistical analyses were performed using GraphPad Prism (version 8.0.2), and RStudio (version 2023.03.1 + 446) and denoted in the figure legends. The χ² test was used to examine differences between patients with and without the NUDCD1 variant, as well as differences between patients with severe disease versus those who did not. To examine the relationship of the NUDCD1 genotype on disease severity, a logistic regression model was fitted using stepwise variable selection for the outcome of oxygen requirement. We included NUDCD1 genotype as well as clinical features of age, sex, ethnicity, and the presence of chronic pulmonary disease as model covariates. Statistical significance was defined as P<0.05.

## Results

### Whole exome sequencing identified NUDCD1 variant which increased likelihood of COVID-19 disease severity

We performed WES on 136 COVID-19 patients in Singapore as described in **Table 2**, of whom 95 (69.9%) were males and mean age was 45.8 years (range 32-57). The patients recruited here are representative of the Asian ethnic distribution of Singapore. Thirty-four of the patients (25%) had severe COVID-19 disease based on requirement for oxygen supplementation or ventilation, of whom 14 (10.3%) required ICU admission. Presenting symptoms, treatment received, length of stay are as described in **Table 2**. There were no mortalities.

**Table 2:** Patient demographics, presentation and outcomes stratified to NUDCD1 genotype.

	ALL	NUDCD1-WT/WT	NUDCD1-WT/V	NUDCD1- V/V
N	136 (100%)	25 (18.4%)	64 (47.0%)	47 (34.6%)
Basic Demographic				
Age, median (IQR)	45.8 (32, 57)	52 (35, 64)	44.5 (31.8, 56)	44 (30, 57)
Sex, Male (n, %)	95 (69.9%)	16 (64.0%)	44 (68.8%)	35 (74.5%)
Ethnicity				
Chinese, n (%)	78 (57.3%)	17 (68.0%)	37 (57.8%)	24 (51.1%)
Malay, n (%)	10 (7.4%)	2 (8.0%)	4 (6.3%)	4 (8.4%)
Indian, n (%)	14 (10.3%)	2 (8.0%)	7 (10.9%)	5 (10.6%)
Others, n (%)	34 (25.0%)	4 (16.0%)	16 (25.0%)	14 (21.9%)
Initial Presentation				
Fever, n (%)	100 (73.5%)	20 80.0%)	43 (67.2%)	37 (78.7%)
Cough, n (%)	87 (64.0%)	19 (76.0%)	42 (65.6%)	26 (55.3%)
Sore throat, n (%)	59 (43.4%)	11 (44.0%)	31 (48.4%)	17 (36.2%)
Outcome				
Oxygen requirement, n (%)	34 (25.0%)	7 (28.0%)	7 (10.9%)	20 (42.6%)
Discharge alive, n (%)	136	25 (100%)	64 (100%)	47 (100%)
Treatment Received				
Steroids, n (%)	1 (0.7%)	0 (0%)	1(1.6%)	0 (0%)
Anti-Viral, n (%)	43 (31.6%)	8 (32.0%)	16 (25.0%)	19 (40.4%)

**Figure 1:**
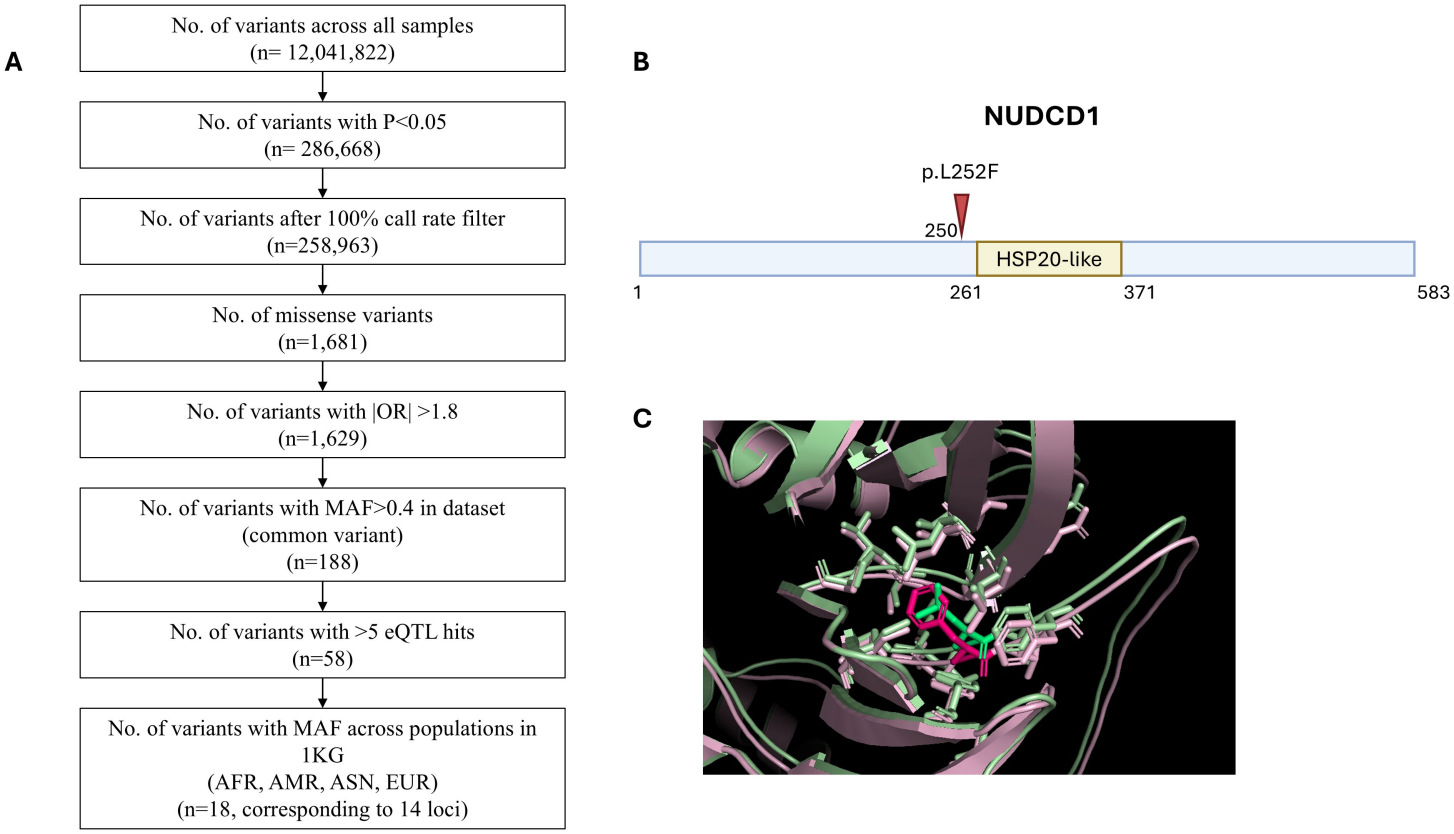
Whole exome sequencing identifies NUDCD1 polymorphism in influencing COVID-19 disease severity. **(A)** Filtering strategy. **(B)** Schematic representation of the p.L252F mutation (arrow) in the NUDCD1 amino acid sequence. Putative HSP20-like domain of NUDCD1 from InterPro (https://www.ebi.ac.uk/interpro/). **(C)** Superimposed protein structures of the wild type NUDCD1 and p.L252F variant.

We performed logistic regression using endpoint of requiring oxygen supplementation in PLINK, adjusting for age and gender. We started with 12,041,822 variants across all patients, with 258,963 variants remaining with P<0.05 and after 100% call rate filter. Prioritisation of missense variants with odds ratio (OR) greater than 1.8 narrowed the field to 1,629 variants. Our aim had been to identify common variants with potential wider applicability to the study population and targeting gene expression in association with multiple gene sets implying potential key biological activity. With these intents, applying a MAF>0.4 in the dataset filtered down to 188 common variants and further selection of those with >5 eQTL hits had led to 58 remaining variants eventually. For practicality, a final filter pointing to potential applicability to worldwide population was applied: we further selected variants with MAF>0.4 across diverse ethnic 1KG cohorts (AFR, AMR, ASN, EUR). This narrowed the list to 18 variants across 14 loci. **Figure 1A** outlines the number of variants remaining at each step of filtering process. The SNP rs2980619 belonging to NudC Domain Containing 1 (NUDCD1) emerged as a shortlisted gene variant of interest. NUDCD1 is ubiquitously expressed in the body, including the lungs and immune cells (30, 31). It lies on exon 5 of chromosome 8, position 110302047, with thymine (T) as its reference base for the wild type (WT) allele. The alternative base known from Genome Aggregation Database (gnomAD) is guanine (G) for the variant (V) SNP; the primary reference T allele frequency is 41.8% and primary alternative G allele frequency is 58.2%. The switch of T-to-G results in an amino acid change from leucine (L) to phenylalanine (F) at position 252 (p.L252F) of the mature protein (**Figures 1B, C**).

In the study population, 18.4%, 47.0% and 34.6% of the patients were homozygous WT(L252)/WT(L252), heterozygous WT(L252)/V(p.L252F) and homozygous V(p.L252F)/V(p.L252F), respectively, for NUDCD1. Further stratification of the patient demographics and clinical status based on the respective NUDCD1 genotypes (WT/WT, WT/V, V/V) are detailed in **Table 2**. Univariate analysis revealed the NUDCD1 V/V genotype, age >50 years old, and male gender to be linked to severe disease (**Table 3**). Possession of the homozygous V/V genotype increased the odds of severe disease by 3.97x (95% confidence interval [CI] 1.76-8.94, P*<* 0.001). Age >50 years and male sex were significant covariates which increased the odds of severe disease by 3.38x (95% CI 1.48-7.68, P=0.003) and 3.16x (95% CI 1.13 – 8.88, P=0.036), respectively (**Table 3**). Ethnicity differences and the presence of chronic pulmonary comorbidities were not statistically significant in the model.

**Table 3:** Logistic regression with stepwise fitting of NUDCD1 genotype, age, gender and presence of chronic pulmonary diseases as covariates for risk estimates and odds ratio of severe COVID-19 disease.

	Non-severe	Severe	*p*-value
**Genotype, *n* (%)** Wild type, WT/WT Heterozygous variant, WT/V Homozygous variant, V/V	18 (17.6) 57 (55.8) 27 (26.5)	7 (20.6) 7 (20.6) 20 (58.8)	< 0.001
**Age, *n* (%)** < 50 years > 50 years	63 (61.8) 39 (38.2)	11 (32.4) 23 (67.4)	0.005
**Gender, *n* (%)** Female Male	36 (35.3) 66 (64.7)	5 (14.7) 29 (85.3)	0.040
**Ethnicity, *n* (%)** Chinese Malay Indian Other	60 (58.8) 5 (4.9) 10 (9.8) 27 (26.5)	18 (52.9) 5 (14.7) 4 (11.7) 7 (20.6)	0.269
**Genotype, *n* (%)** Wild type or heterozygous variant, WT/WT or WT/V Homozygous variant, V/V	75 (73.5) 27 (26.5)	14 (41.2) 20 (58.8)	0.001

Variable	Estimate (SE)	Odds ratio (95% CI)	*p*-value
**NUDCD1 Homozygous variant**	1.59 (0.47)	3.97 (1.76 – 8.94)	< 0.001
**Age** ≤ 50 years > 50 years	1.58 (0.47)	3.38 (1.48 – 7.68)	0.003
**Gender** Male	1.27 (0.60)	3.16 (1.13 – 8.88)	0.036
**Ethnicity** Other Chinese Malay Indian	-0.11 (0.64) 0.67 (0.88) 0.09 (0.83)	1.16 (0.43 – 3.10) 3.86 (0.87 – 17.16) 1.54 (0.37 – 6.43)	0.861 0.449 0.910
**Chronic pulmonary disease**	0.01 (1.31)	0.74 (0.08 – 6.88)	0.767
**Intercept**	-3.39 (0.83)		< 0.001

### NUDCD1 p.L252F variant resulted in reduced protein expression and attenuated activation of DHX15 and MAVS

**Figure 2:**
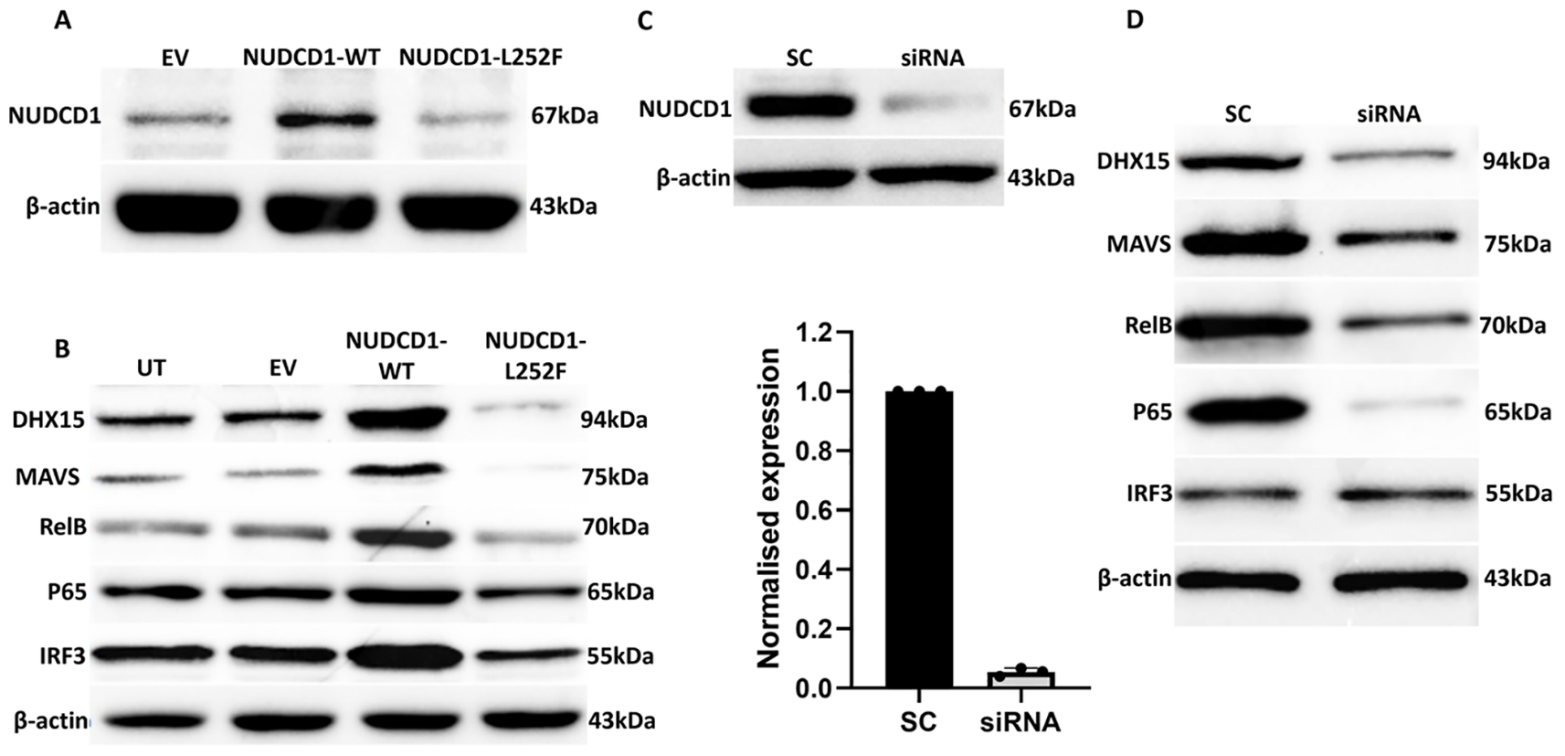
The NUDCD1 p.L252F variant results in reduced protein expression and attenuated activation of DHX15 and MAVS. **(A)** Diminished levels of NUDCD1 in NUDCD1 p.L252F-transfected A549-ACE2 cells compared to wild type NUDCD1-transfected cells. β-actin was used as a loading control. **(B)** Reduced protein levels of DHX15, MAVS, RelB and p65 in NUDCD1 p.L252F-transfected A549-ACE2 cells. IRF3 levels remained similar between the wild type and p.L252F NUDCD1 variant. β-actin was used as a loading control. UT: untransfected; EV: empty vector. **(C)** Efficiency of NUDCD1 siRNA knockdown determined by Western blot and qRT-PCR. **(D)** Reduced protein levels of DHX15, MAVS, RelB and p65 in NUDCD1 knockdown. β-actin was used as a loading control. SC: scrambled siRNA, negative control.

NUDCD1 is located in the cytosol and is thought to be involved in modulation of the immune system process and linked to autoimmune atherosclerosis (32). This gene and its protein function are not understood in the context of COVID-19 or infections per se. To study its role in disease causation, WT NUDCD1 (L252) and its non-synonymous variant carrying the alternative allele G (NUDCD1 p.L252F) were cloned and transfected into A549-ACE2 cells. Cells transfected with the NUDCD1 p.L252F variant showed diminished NUDCD1 expression compared to WT NUDCD1 (L252) (**Figure 2A**).

Recently, NUDCD1 was reported to have a putative relationship with the RNA helicase DEAH-Box Helicase 15 (DHX15) (33). The latter is involved in defence against RNA viruses which is mediated through MAVS (Mitochondrial Antiviral Signalling Protein) (34). MAVS is critical in triggering type I interferon response against viruses including Coronaviruses (35). The convergence of these findings, yet to be reported, may explain a role of NUDCD1 in COVID-19 disease.

We demonstrated that WT NUDCD1 (L252) had higher NUDCD1 protein expression than NUDCD1 p.L252F. In turn, WT NUDCD1 (L252)-transfected cells induced greater expression of DHX15 and MAVS than variant NUDCD1 p.L252F cells. Given that the induction of innate antiviral response by MAVS is through NFκB, we investigated whether components of the NFκB complex requisite for cytokine production were affected. Our results demonstrated that the alternative NUDCD1 p.L252F variant led to reduced activation of NFκB components RelB and p65 (**Figure 2B**). In contrast, IRF3 was less affected by altered NUDCD1 function.

To validate the consequence of reduced NUDCD1 activation such as in the setting of NUDCD1 p.L252F, we utilised siRNA knockdown against NUDCD1 in A549-ACE2 cells. Effective silencing of NUDCD1 (**Figure 2C**) resulted in diminished induction of DHX15 and MAVS, and similarly reduced RelB and p65 expression (**Figure 2D**), as observed with NUDCD1 p.L252F cells. Taken together, our findings suggest that the diminished NUDCD1 activity by the p.L252F variant resulted in decreased effector antiviral response against SARS-CoV-2.

### NUDCD1 p.L252F cells show increased susceptibility to infection by SARS-CoV-2 and reduced cytokine production

**Figure 3:**
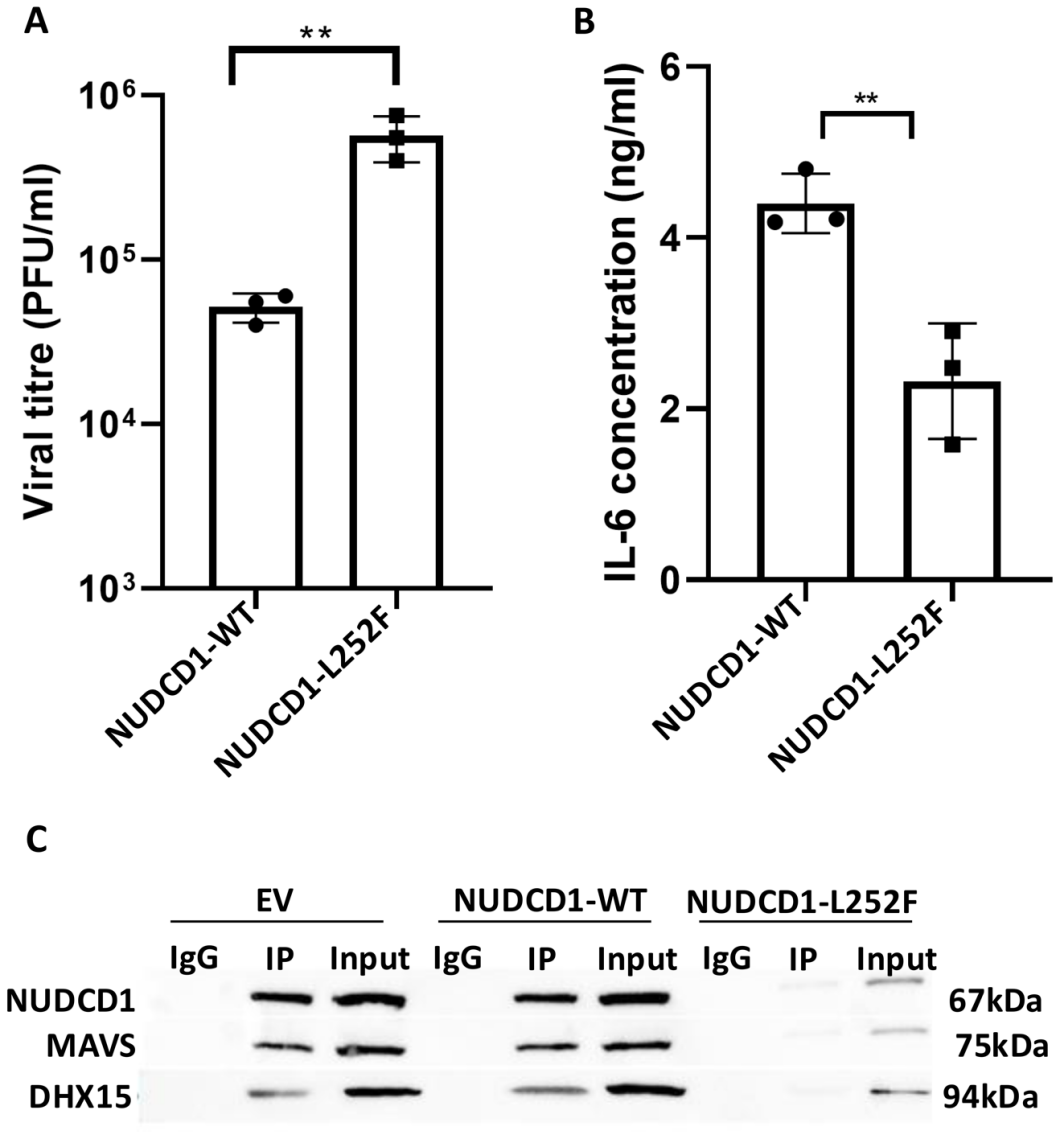
NUDCD1 p.L252F cells show increased susceptibility to infection by SARS-CoV-2 and reduced interleukin 6 production. **(A)** SARS-CoV-2 viral load from A549-ACE2 cells transfected with plasmids expressing either the wild type or p.L252F NUDCD1 variant (n=3). Statistical significance was performed with unpaired t-test. **: P<0.01. **(B)** Interleukin 6 levels from SARS-CoV-2 infected cell supernatant (n=3). Statistical significance was performed with unpaired t-test. **: P<0.01. **(C)** Association of NUDCD1 and MAVS with DHX15 by co-immunoprecipitation from SARS-CoV-2 infected cell lysates.

To elicit the virologic sequelae of altered NUDCD1 function in its susceptibility to SARS-CoV-2 infection, we transfected A549-ACE2 cells with plasmid expressing either WT NUDCD1 (L252) or variant NUDCD1 p.L252F and infected the cells with the SARS-CoV-2 virus. We observed that cells transfected with WT NUDCD1 (L252) were more resistant to SARS-CoV-2 than the NUDCD1 p.L252F variant. The NUDCD1 p.L252F-transfected cells sustained significantly higher viral load by approximately 1-log10 compared to WT NUDCD1 (L252)-transfected cells (**Figure 3A**). In addition, we observed that NUDCD1 p.L252F cells produced significantly lower levels of IL-6 compared with WT NUDCD1 (L252) cells (**Figure 3B**), in line with findings of reduced MAVS and RelB/p65 activation in NUDCD1 p.L252F cells (**Figure 2B**). Using infected cell lysates, co-immunoprecipitation of DHX15 with NUDCD1 and MAVS demonstrated that in NUDCD1 p.L252F-tranfected cells, there were decreased NUDCD1 and MAVS interaction with DHX15 (**Figure 3C**). These results suggest that the NUDCD1 p.L252F variant leads to impairment of NUDCD1 function through its decreased interaction with DHX15 and MAVS, resulting in increased SARS-CoV-2 susceptibility.

### Patients bearing NUDCD1 p.L252F have reduced NUDCD1 expression

**Figure 4:**
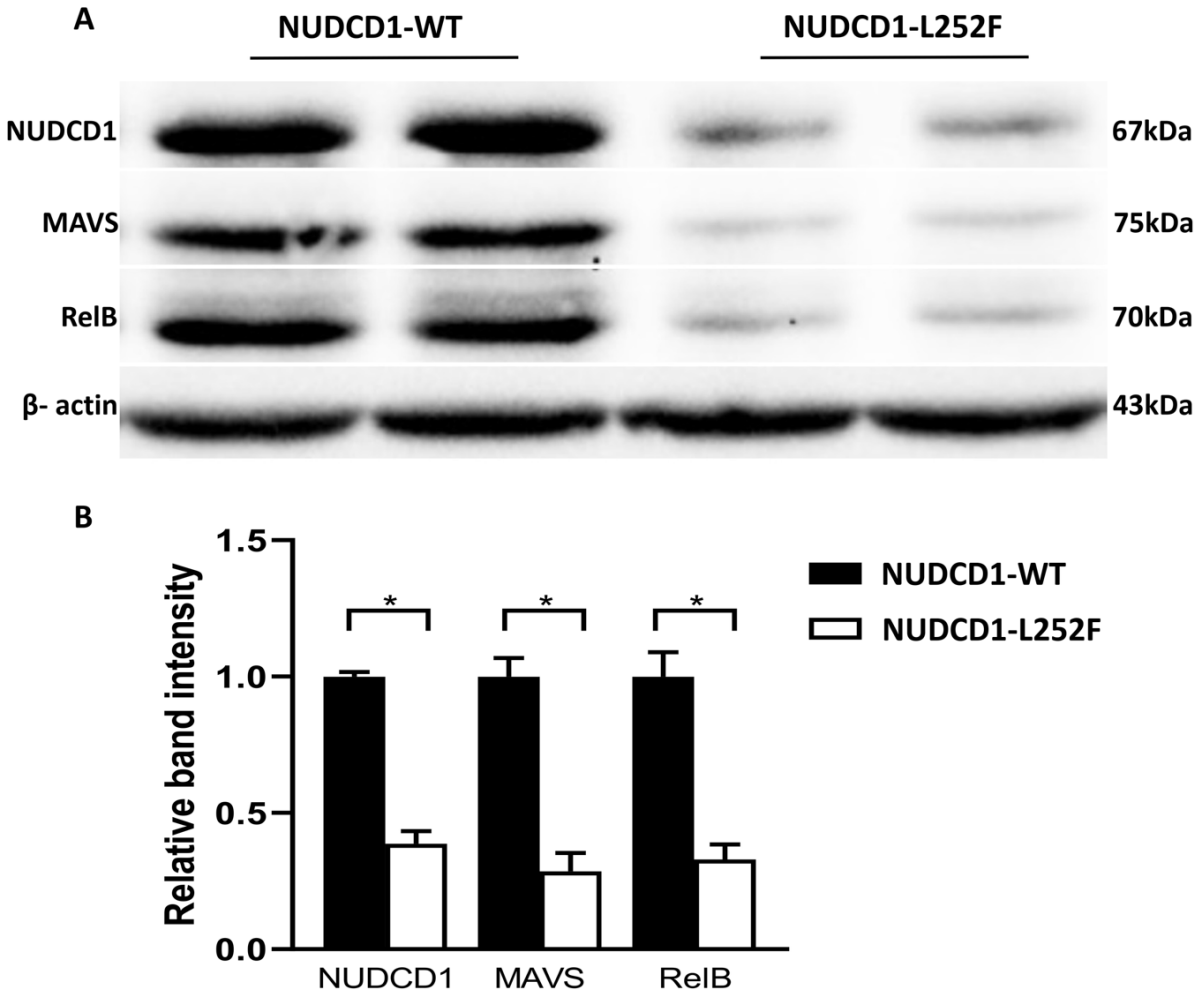
Patients bearing the NUDCD1 p.L252F variant have reduced NUDCD1 protein expression. **(A)** Diminished NUDCD1 expression from patient PBMCs with the p.L252F variant. Likewise, MAVS and RelB were attenuated. β-actin was used as a loading control. **(B)** Respective densitometric analysis of western blot result. Western blots were normalised to β-actin and densitometric analysis was performed using image processing software. Values are mean ± standard error of the mean of L252 (n=4) and p.L252F (n=5). Statistical significance was performed using Mann-Whitney U test. *: P<0.05.

To re-validate the above findings, PBMCs from SARS-CoV-2-infected patients who bore WT NUDCD1 (L252) or variant NUDCD1 p.L252F were tested. Notably, cells of patients with NUDCD1 p.L252F showed marked reduction of NUDCD1, MAVS and RelB expression (**Figures 4A, B**), in line with the *in vitro* findings.

Overall, the infection studies align with findings from genetic and mechanistic studies. WT NUDCD1 (L252) is protective against severe COVID-19. A non-synonymous switch from T to alternative G led to altered protein function in NUDCD1 p.L252F, which compromised upfront antiviral effector response through DHX15, MAVS and NFκB complex. This diminished innate response hampered effective clearance of virus and resulted in higher SARS-CoV-2 viral load and consequently more severe COVID-19 disease.

## Discussion

The distinction of true disease-causing variants from the numerous potentially functional variants derived from genomic association is critical (36). With high throughput data arising from current genomic arrays and sequencing platforms, assignment of causality through demonstration of direct evidence of pathogenicity is all the more vital to weed out false-positive variants which can be retained despite the varied filtering strategies (37). Conversely, potential true disease-causing variants may be missed out in the absence of a fully or even intermediate penetrant phenotype, or if the study sample size, be it on resource-restricted or minority grounds, fails to attain the power of genetic homogeneity and statistical strength (21, 38). This calls for additional tiers of evidence to demonstrate the damaging (or protective) impact of genetic variants in disease pathophysiology. The guidelines by MacArthur proposed that genetic associative findings of gene variants significantly enriched in cases compared with controls be strengthened by informatic *in silico* data and very importantly, experimental evidence (36, 39). Experimental studies ought to demonstrate biologically-plausible causal role of the specified variant through techniques involving gene disruption and manoeuvres to recapitulate the observed phenotype in *in vitro*, ex vivo, animal experimental setting or as applicable, in the patient cohort of interest. We had sought to fulfil these requisite criteria to link molecular epidemiology to pathogenesis with emphasis on validation given that this had not been widely attempted in the context of severe COVID-19 disease.

Our WES output had yielded NUDCD1 rs2980619 as a top candidate amongst enriched variants which emerged from comparing patients with severe and mild COVID-19 disease. There was an odds ratio difference of 3.97x higher likelihood of severe disease in subjects homozygous for NUDCD1 p.L252F, of which the reference allele was protective. This lesser known gene was first described in the context as a tumour antigen (also known as Chronic Myelogenous Leukemia Tumour Antigen 66 [CML66]), although it has been perceived as being an immunogenic epitope capable of evoking host immune response (40, 41). Besides carcinogenesis, the parental NUDC (Nuclear Distribution Protein C) has been shown to be involved in haematopoiesis, cell cycle progression and inflammation (42). NUDCD1 is now known to be ubiquitously expressed in almost all major organs and particularly at higher levels in white blood cells and lung tissues as seen on both mRNA and proteomics arrays as well as in ciliated epithelium of bronchial mucosa (43–46). The viability of NUDCD1’s involvement in COVID-19 disease was supported by prior description of NUDCD1 as a high-placed candidate amongst a catalogue of genes listed in a COVID-19 severity association study though this track was not further pursued, as well as its described link with an RNA helicase DHX15 with ascribed antiviral role (33, 47).

The consequential non-synonymous allele switch from T to G results in the amino acid substitution p.L252F, leading to decreased expression of the protein. Furthermore, p.L252F lies (5 amino acids) proximal to the HSP20-like chaperon domain of NUDCD1 (48). HSP20 as a family of small heat shock proteins (sHSP) serve as molecular chaperons, which are activated to mitigate states of molecular stress through its multiple regulatory roles within the nucleus including inhibition of caspase-3 and apoptosis (49, 50). This molecular chaperon function of the NUDC family is further extended through the demonstration of its interaction with DEAD/DEAH box helicases, and more specifically recently, co-association studies showing unique NUDCD1 interaction with DHX15, a DEAD/DEAH box helicase (33, 51). In our study, we further demonstrate the interaction of NUDCD1 with DHX15 experimentally through co-immunoprecipitation. The attenuated NUDCD1 expression from p.L252F led to diminished DHX15 and MAVS activities. DHX15 is a well-recognised viral RNA sensor essential for type I interferon which can trigger MAVS, a critical adaptor for virus-induced signalling through NFκB (34, 52). This interaction with DHX15 and MAVS was replicated by our targeted silencing of NUDCD1.

**Figure 5:**
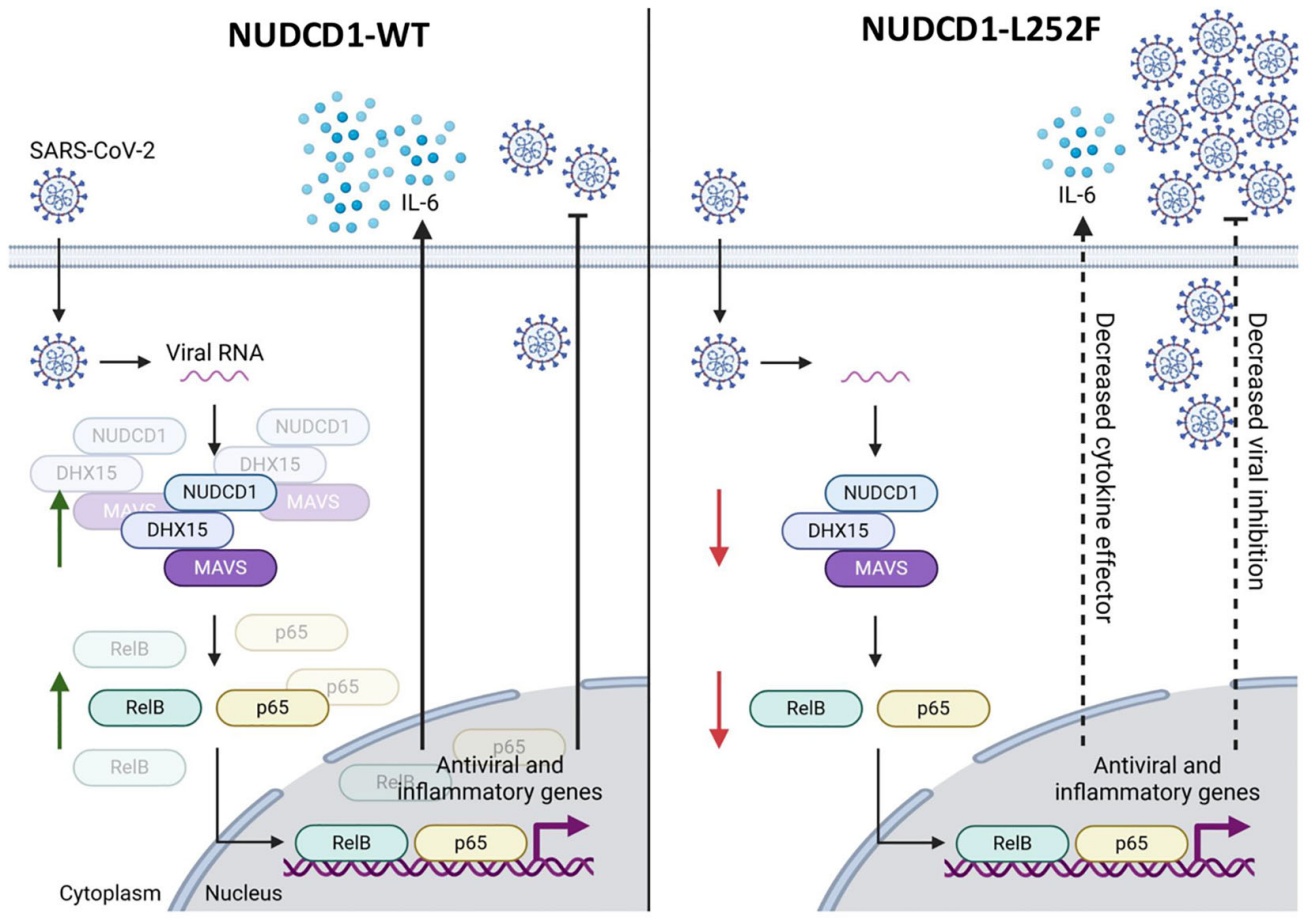
The NUDCD1 p.L252F variant impairs antiviral signalling and cytokine response during SARS-CoV-2 infection. This schematic illustrates the differential immune response to SARS-CoV-2 infection mediated by wild type NUDCD1 (left) and variant NUDCD1 p.L252F (right). (Left) Upon SARS-CoV-2 infection, viral RNA is detected in the cytoplasm by the protein complex involving NUDCD1, DHX15, and MAVS. This complex facilitates the activation of transcription factors RelB and p65, which translocate into the nucleus and drive the expression of antiviral and inflammatory genes, including IL-6. The result is effective cytokine signalling and viral inhibition. (Right) The variant impairs the assembly or function of the NUDCD1-DHX15-MAVS complex, reducing the activation of RelB and p65. This leads to lower cytokine effector function, as exemplified by decreased IL-6 and reduced viral inhibition, allowing for increased viral replication. Green arrows indicate upregulation or activation, while red arrows indicate downregulation or impaired signalling. Created with www.biorender.com.

We strengthened these mechanistic findings to the SARS-CoV-2-infected patients whereby bearers of NUDCD1 p.L252F had indeed lower NUDCD1, MAVS and RelB expression compared with those who were homozygous for the WT allele. The patients had been recruited prior to introduction of SARS-CoV-2 immunisation *v.i.z.* the immune trait elicited was independent of the vaccine effect. As much as may be speculated if effective vaccination might mitigate against severe COVID-19 disease, this unique unvaccinated study cohort provides us with the opportunity to study host susceptibility to SARS-CoV-2 infection and disease process unadulterated. Further to the widely held perception directly linking the said hyperinflammatory phase of SARS-CoV-2 infection to severe disease, our findings rather, suggest that the critical determinant of severe disease is the ability of the host immunity to mount an adequate immune response upon early encounter with the SARS-CoV-2 virus. As exemplified by the hypomorphic NUDCD1 p.L252F variant and the compromised antiviral effectors DHX15 and MAVS, the resultant diminished proinflammatory cytokines and type I interferon facilitated SARS-CoV-2 virus proliferation. Our findings here are as schematically summarised in **Figure 5**. In turn, it is the higher SARS-CoV-2 viral burden which triggers the host immunity to an exaggerated hyperinflammatory response.

Indeed, over the years of this pandemic, we have now a better understanding that different therapeutics are to be deployed for the different phases of SARS-CoV-2 infection: use of antivirals for early infection, and immune modulators like steroids in the later ensuing hyperinflammatory phase. Our study supports the stance of adopting an antiviral and immune-augmenting strategy at the earliest opportunity following infection by the SARS-CoV-2 virus. This has been as demonstrated by use of the antiviral remdesivir (53) or the immunostimulatory pegylated interferon lambda (54) in early COVID-19 infection which led to reduced hospitalisation risks. And yet in another major clinical study of patients at high risk of severe disease progression, early combination remdesivir and interferon beta-1b treatment shortened viral shedding, alleviated symptoms faster and reduced hospitalisation (55).

SARS-CoV-2 infections will persist in the years to come. In tandem with ongoing epidemiological interests in chasing the dynamic evolution of the circulating virus strains, continued studies to better understand the other critical aspect determining disease outcome *v.i.z.* the host and underlying traits predisposing to severe disease are important. This is achieved through understanding pathogenesis to guide development of new treatment or management strategies including genetic stratification tests, which may be less challenging and more fruitful than chasing a moving target with monoclonal antibody development against a continuously evolving virus. Of the discovery studies to date on the genetic factors affecting SARS-CoV-2 infection risk which have been mainly associative in method, we have elicited a novel gene variant of NUDCD1 in Asian patients which influences disease severity. This has been validated mechanistically to highlight its functional role and disease causation.

## Data Availability

The data presented in the study are deposited in the NCBI Sequence Read Archive (SRA) under BioProject ID PRJNA1266378, and are available at the following link: https://www.ncbi.nlm.nih.gov/bioproject/PRJNA1266378.
